# Epistemological and ethical assessment of obesity bias in industrialized countries

**DOI:** 10.1186/1747-5341-6-16

**Published:** 2011-12-16

**Authors:** Jacquineau Azétsop, Tisha R Joy

**Affiliations:** 1 Département de Santé Publique, Faculté des Sciences de la Santé de l'Université de N'djaména, Avenue Mobutu, B.P. 1117 N'djaména, Tchad; 2 Department of Medicine and Dentistry, Division of Endocrinology and Metabolism, University of Western Ontario, B5-107, 268 Grosvenor Street, London, Ontario, N6A 4V2, Canada

## Abstract

Bernard Lonergan's cognitive theory challenges us to raise questions about both the cognitive process through which obesity is perceived as a behaviour change issue and the objectivity of such a moral judgment. Lonergan's theory provides the theoretical tools to affirm that anti-fat discrimination, in the United States of America and in many industrialized countries, is the result of both a group bias that resists insights into the good of other groups and a general bias of anti-intellectualism that tends to set common sense against insights that require any thorough scientific analyses. While general bias diverts the public's attention away from the true aetiology of obesity, group bias sustains an anti-fat culture that subtly legitimates discriminatory practices and policies against obese people. Although anti-discrimination laws may seem to be a reasonable way of protecting obese and overweight individuals from discrimination, obesity bias can be best addressed by reframing the obesity debate from an environmental perspective from which tools and strategies to address both the social and individual determinants of obesity can be developed. Attention should not be concentrated on individuals' behaviour as it is related to lifestyle choices, without giving due consideration to the all-encompassing constraining factors which challenge the social and rational blindness of obesity bias.

## Introduction

Although bias can lead to discrimination, the two concepts do not have the same meaning. Bias denotes a judgment without sufficient knowledge, while discrimination is the process by which two stimuli differing in a single aspect are responded to differently. Bias refers to attitudes while discrimination refers to a given behavior. Most often, these two terms evoke associations with race or gender. In such associations, one race or gender is seen to be superior to another. Popular culture has been inundated with biased attitudes toward obese individuals. These people are described as lazy, gluttonous, or stupid. Such attitudes give rise to jokes and sarcasm about overweight or obese individuals. The lack of will as the causal attribution for obesity and societal obsession with being thin has been central to the development of anti-fat bias [1]. It has been documented that overweight/obese individuals experience anti-fat bias and discrimination in their academic, family, social, healthcare, and employment settings [2]. Sadly, even children as young as three have been known to exhibit bias against overweight individuals even though they are not able to express properly their thought at that age [3]. Anti-fat obesity is of one of the most acceptable forms of bias [4].

This paper adheres to Bernard Lonergan's cognitive theory in an attempt to reject the biases leading to an unbalanced understanding of obesity. We will demonstrate that such an understanding of obesity lacks epistemological and ethical validity. We also argue for framing obesity within an environmental model. Such a model makes a good arena to pose questions about biases that shape policy and government intervention personal discrimination against obese individuals and socioeconomic exclusion that increase risks for obesity. Following Lonergan's cognitive theory, we are challenged to raise questions about both the cognitive process through which obesity is perceived as a behavior change issue and the objectivity of such a moral judgment. We also show that anti-fat discrimination, in many industrialized countries, is the result of a general bias of anti-intellectualism that tends to set common sense against insights that require scientific investigation analysis. The lack of deep analyses and sound judgments often sustain the group bias that resists insights into the good of other groups.

While general bias diverts public attention away from the distal causal factors for obesity, group bias sustains an anti-fat culture that subtly legitimates discriminatory practices and policies against obese people. These practices and policies are, whether implied or stated, a rash judgment about a person's reason for being obese. When they exist, anti-fat discrimination laws narrowly portray obesity as a problem pertaining to the individual whose rights need to be protected. Although anti-discrimination laws may seem to be a reasonable way of protecting obese and overweight individuals from stigmatization and discrimination, this paper proposes that obesity bias can be best addressed by reframing the obesity debate in an environmental perspective from which tools and strategies can be developed to address both the distal and proximal determinants of obesity. Reframing obesity from a public health perspective will give a comprehensive view of the causes and impacts of being obese. Situating the problem of obesity in the general setting of public health will also help to elucidate the problem of a socioeconomic exclusion that increases the risk for obesity among lower socioeconomic groups. Attention is often focused on the narrow dimensions of individual behavior change as it is related to lifestyle choices. Such focus does not give due consideration to the all-encompassing constraining factors in an environment of economic exclusion. These factors include, but are not limited to: material deprivation, racial and neighbourhood segregation, food pricing, the high concentration of fast food vendors in poor neighbourhoods, dietary habits, impacts of food advertising on diet, and features of the built environment that influence individual lifestyle and dietary habits. The built environment refers to the man-made surroundings that provide the setting for human activity, ranging in scale from personal shelter to neighborhoods to the large-scale civic surroundings. The shape of the man-made environment and all the other remote causal factors for obesity challenge the social blindness of the general bias of anti-intellectualism and raise ethical questions concerning the justice of social institutions and policies which are shaped by economic individualism that produces group bias.

## Epidemiological Evidence of Anti-Fat Biases and Discrimination

Although weight-based teasing in adolescence seems to be very common [5], data regarding the actual prevalence of anti-fat bias and discrimination in adults is minimal. Perceived weight-to-height discrimination has increased by ~66%, from 7% in 1995-1996 to 12% in 2004-2006. These results were based on surveying a representative sample of US adults aged 35-74 years [6]. Significant predictors of this perceived discrimination have included being female and/or having increased body mass index within a certain range [7]. In absolute values, the prevalence of perceived weight-to-height discrimination among the overweight, moderately obese, and severely obese was 6.9%, 14.2%, and 42.5%, respectively, in 2004-2006 [6].

A study showed that anti-fat discrimination can take many forms and can be perpetrated by both children and adults. Children, independent of their own age, gender, and weight, demonstrate negative stereotypes for not only obese children but also obese adults [8]. Children's perception of obese individuals was very negative compared to their perception of non-obese. The obese child/adult was more likely to be rated lazy and less likely to be rated attractive, confident, or happy. These children were also more likely to choose the child who was normal-weight rather than the obese one for their friend or playmate. Interestingly, children uniformly believed that obesity was under individual control. The degree of controllability assigned to obesity by the children was directly correlated with negative stereotyping [8]. Similar negative views are often held by teachers and educators with obese students described as more emotional, less likely to succeed at work, or to have more family problems compared to students who were not obese [9]. Forty-two percent of teachers perceived obese individuals as less sexually attractive than non-obese individuals and forty seven percent strongly agreed that most individuals who were not obese would not want to marry an obese individual [9]. These results echo the typical portrayals of overweight/obese characters seen on television [10]. Overweight or obese female characters were less likely to be physically affectionate, romantically involved, or considered attractive while overweight or obese male television characters were more often seen eating and less likely to be romantically involved [10].

In 1966, Canning and Mayer demonstrated that overweight/obese adolescents were less represented at several prestigious colleges and universities in the United States despite having relatively similar academic achievements [11]. Further studies have demonstrated that heavy-weight college students, particularly female, have to rely on non-familial sources of support for college education compared to students with normal weight [12]. This lack of parental financial support was strongest for daughters of political conservatives, a group found to endorse anti-fat attitudes [13]. Some schools have even gone as far as mandating weight loss and dismissing students if weight loss is not achieved [14]. In some of these cases, obese applicants were less likely to be contacted and/or hired compared to normal weight applicants despite both having similar resumes [14,15]. Although the faces of the applicants were never seen and an off-camera voice was used as a constant in all settings, the obese applicant was viewed as having poorer work habits due to misuse of time. The obese applicants were also viewed as having emotional and interpersonal issues [16]. Similar findings of hiring discrimination exist even when average-weight applicants were dressed in prostheses to mimic overweight applicants [17].

Anti-fat bias has been demonstrated to be significantly greater than other forms of bias [18]. Moreover, individuals are often willing to either forego positive events or endure negative events to attain or maintain a non-obese weight [19,20]. Schwartz et al demonstrated that of all respondents, ~46% were willing to give up one year of life and 15% were willing to give up ten or more years of life rather than be obese [21]. These personal sacrifices were significantly influenced by BMI, with underweight respondents demonstrating a higher degree of willingness to make personal sacrifices than extremely obese respondents. For example, although only 5% of respondents overall were willing to lose a limb rather than be obese, this value was 22.4% in underweight respondents and 3.0% in the extremely obese respondents [21]. Although the overall percentage of respondents willing to make child-related trade-offs was lower than that for personal sacrifices, still 10% and 8% of respondents would rather have an anorexic child or learning disabled child, respectively, rather than an obese child [21]. Similar to the findings related to personal sacrifices, the responses for child-related trade-offs were significantly influenced by BMI [21]. Although much work remains to further delineate the prevalence of anti-fat bias, the data far demonstrate that discrimination, and the stigma associated with being obese are present to a large degree in society.

## Epistemological Assessment of General Bias

General bias raises epistemological concerns. These epistemological concerns emerge from logical assumptions that connect empirical experience to human cognitional structure. The cognitive process leads to normative judgments and policy decisions on obesity. How do people reach the conclusion that obesity is an issue of personal virtue or social justice? How do they move from the empirical facts regarding the genetic, social, and environmental determinants of obesity to endorse negative stereotypes that encourage anti-fat attitudes? Meanwhile, group bias raises ethical concerns. These ethical concerns question how knowledge about weight management determines public perceptions and policies as well as individual attitudes of discrimination.

### General bias raises epistemological concerns

In *Insight*, Bernard Lonergan develops his general empirical method as the basis for making objective judgments and decisions [22]. In order to determine if it is ever possible to make an objective decision, Lonergan first asks, what am I doing when I make a decision [22]? The answer to this question is a theory of knowing which forms the basis of Lonergan's entire philosophy and underlies his answer to the problem of objective decision making. Even after knowing the alternatives, an individual must still decide between them. Lonergan further explores what choosing adds to knowing. Building on this structure, he explains what error is, how it can and does distort the process of decision making in practice. Lonergan also shows how error can be corrected and avoided. Finally, he answers the question directly, and delineates a method for making objective judgments. Human knowing is an activity which intimately connects the knower and the object to be known. This activity occurs through a three-fold structure which includes experience, insight, and judgment [22].

By basing all his work on his understanding of cognitional structure, Lonergan describes the process of knowing from its beginnings in experience. He traces the transformation of experience into understanding through insight and the judgment that asks if the insight is true or not. Finally, he incorporates this structure of knowing into a broader structure of decision which includes determining the value of alternate courses of action, and committing to bring the most valuable alternative into being.

### Experience

A direct or indirect encounter with the object is the precondition of a true knowledge of a thing [22]. Even though the cognitive representation of the object does not always truly represent the object *per se*, experience of the object is important to achieve a certain level of objectivity. Instead of seeking a high level of objectivity, the subject can simply consider his or her own perception of the object as absolute or rely on opinions as the real source of knowledge. A true knowledge of obesity presupposes an objective recognition of all the features and dimensions of weight excess. However, the empirical appraisal of weight excess is often determined by dominant ideologies that assign a moral significance to obesity. These ideologies are essentially based on the individual ability to avert obesity. These ideologies are biased to individualism because they cannot account for the biological, behavioral, and psychosocial mechanisms that determine risk for obesity. Thus, a direct or an indirect experience of obesity, informed by an in-depth scientific investigation is needed to avoid biased judgment. Ideally, however, experience provides the data that allow for the possibility of an insight [22].

### Insight

Insight represents an act of intelligibility. Such an act allows the human mind to grasp information which was previously available but unintelligible. Typically, insight is gained from adherence to the norms of attentiveness, intelligence, reasonability, and responsibility. When these norms inform the process of knowing, relevant questions are raised, coherent sets of insights are assembled, rash judgments are avoided, and the link between judgment and decision-making can be tested. An insight is an answer to a question of reflection: what is obesity and how does it happen? Or it can be an answer to question of intelligence: why does it happen? An insight is the property of a subject and not of the object. However, the subject is never totally free from societal influence or historical determinism. The very act of intelligibility which allows the human mind to understand phenomena and grasp information necessary to make a moral judgement on them can be determined by the dominant social group.

### Judgment

Judgment follows and acts upon insights [22]. Critical reflection and introspection as well as confrontation with other viewpoints are the preconditions for an objective judgment. Objectivity often results from the dialogical synthesis between objective data and subjective acts. With this understanding of judgment, knowing cannot be collapsed into judgment alone. Doing so would simply lead to the perpetuation of the theory that obesity results from controllable factors.

These three parts of the cognitional structure--experiencing, understanding, and judging--all presuppose and complement each other. Experiences without understanding are unintelligible, but understanding requires experience to act upon. Equally, understanding without judgment is meaningless, but judging involves an intimate interaction with an insight, and through the insight, with experience.

## BIAS

Failure to make judgments which are true is caught up in the term "bias" or error. Lonergan describes different forms of biases. These forms of biases include psychological bias such as neurosis that resists insight into one's *psychè and *individual bias of egoism that resists insight into what benefits others. Lonergan's description of biases also include group bias of loyalism that resists insights into the good of other group and general bias of anti-intellectualism that tends to set common sense against insights that require any thorough scientific investigation [22].

Here, our main interest is to demonstrate that anti-fat discrimination and judgments stem both from a general of anti-intellectualism and from a group bias of socioeconomic exclusion.

### Anti-fat bias as a general bias

Lonergan describes the general bias of common sense toward theoretical patterns of knowing. Although the cognitional structure is found in everyone, individual people according to their needs and practices apply it in various situations and towards various ends. There is one very basic distinction in patterns of knowing that causes a great deal of trouble. At certain times, people direct their attention toward scientific, disinterested knowing, and then they anticipate understanding generalizations and technical answers [22]. At other times, people are interested in common sense knowing, in understanding each immediate situation in a very practical way, and seeking particular and practical answers. As even this summary statement of the different purposes of the two patterns of knowing indicates, the two are complementary. Common sense thinking solves practical immediate problems, while theoretical knowing generates long-term planning and describes common-sense situations in helpful ways. The tension generated between the two patterns often breaks down into what Lonergan terms general bias [22]. General bias against theoretical knowing rationalizes as well as blinds. General bias is not a culture but only a compromise that results from taking the highest common factor of an aggregate of cultures [22].

Studies have suggested that general bias is impacted by a social ideology that uses negative attributions to explain negative life outcomes [23,24]. American values of individualism and autonomy provide a foundation for anti-fat attitudes by emphasizing responsibility for one's choice and the willpower to control the circumstances that shape one's life [25]. Both the freedom of choice and willpower are closely related to the values of internal control and self-discipline emphasized by Max Weber in his approach to the protestant work ethic [26]. Max Weber presents hard work as a component of a person's calling. Weber highlights the fact that for Calvinists, worldly success is a sign of personal salvation. Right from the beginning of Protestantism, Martin Luther re-conceptualized worldly work as a spiritual duty which benefits both the individual and society as a whole. Working diligently became a sign of grace. This approach to work emphasizes human agency and freedom to achieve one's goal as a sign of God's blessing. Closely linked to this ethical view, the "just world bias" portrays the world as a predictable environment in which personal effort and ability lead to desired outcomes [27,28]. Those who subscribe to this belief rely on physical attractiveness in making attributional judgments of people [27]. Excess weight in obese individuals is simply perceived as a lack of self-control except when it is associated with a health condition such as thyroid or glandular disorder [29,30]. Moreover, a perceived causality of obesity is highly influential in making stigmatizing attributions [31].

In a study which aimed at measuring stigma toward 66 different diseases and health conditions (including obesity) by 415 participants, Christian Crandall and Moriarty Dallie showed the extent to which the power of attributions of control determines social rejection [32]. The degree to which one is held responsible for the disease or health condition, significantly predicted social distance and rejection by participants [32]. In addition, cross-cultural comparisons of anti-fat attitudes in Mexico and the USA revealed that anti-fat attitudes were strongly associated with socio-ideological variables in the American sample. This relationship was non-existent among Mexicans whose attributions were not connected to social ideologies [25]. The association between social ideologies and anti-fat attitudes were mediated by an attribution of controllability, by which social ideological beliefs were related to belief in autonomy and dislike among American students. However, social ideological beliefs were unrelated to either of these constructs among Mexicans [25]. These results echo those by Crandall et al demonstrating perceived controllability of obesity as an important determinant of general bias [31].

Society's biased perception of obesity has negative influence on individuals' ability to understand the causes of this epidemic. Hence, anti-fat bias can be understood as a general bias of anti-intellectualism because the individual uncritically accepts a cultural viewpoint which is not scientifically accurate. Nevertheless, this view point is commonly accepted across culture and held as the truth. On the other hand, anti-fat bias is a group bias because it does not only result from the resistance to true science but also from socioeconomic exclusion.

### Social ideology as a group bias: questioning the epistemological foundations of group bias

Group bias resists insight into the good of others. Group bias often leads to the marginalization of one group for the good of another. Those who are inclined to uphold this type of bias rely on divisive ideologies based on class, race, nation, weight or gender. Group bias occurs when a social group within a community resist changes, either actively or passively. In doing so, such a group avoids understanding how changes would actually improve the community [22]. Culture is a carrier of great and negative ideologies. Culture, as a whole and its various parts, must be scrutinized against the ideal of a good society in order to identify ideas and practices that prevent individuals or institutions from making objective decisions [22]. Group bias leads to a failure to appreciate others, to nationalism, class hatred, socioeconomic exclusion and ignorance of science or philosophy.

Many Americans question whether it is appropriate for the government to target a non-communicable disease that most individuals could avoid by making healthy lifestyle choices. The rejection of state intervention is often grounded in the affirmation of individual autonomy. This societal approach to autonomy asserts one's freedom to make choices, even wrong ones, so long as they do not harm others. Policy makers respect autonomy because it is a value accepted by many citizens. American health policy choices have historically reflected a preference for markets over government provision of health services, for treatment over prevention, and for expenditures to reduce uncontrollable risks over controllable risks [22]. While there is more talk than ever about an unhealthy environment contributing to obesity, there is less acceptance of the idea that risk has been incurred involuntarily by overweight adults. To absolve individuals of all responsibility for their weight would defy cultural norms and common sense. But even relieving them of some responsibility appears difficult [22]. Obesity is, thus, a private matter and not necessarily a public health issue that society needs to help solve.

Calls for government intervention to prevent obesity and protect obese individuals from discrimination have met with significant socioeconomic and political resistance. There has been an ongoing debate, both philosophically and ideologically charged, between those who stress the importance of the community and those who highlight the importance of individuals' rights. Unfortunately, the growing polarization on individual choice has moved the debate on the individual side of the balance. This individualized understanding of human action has deeply influenced how responsibility to individual infection or pathology is assigned. However, we critique the ideological presupposition of this approach to responsibility for health because it does not question the social construct of health problems. Society can be blinded by an ideology that protects and promotes the welfare of powerful institutions and individuals while ignoring the sufferings endured by the worse off. The cultural resistance to government intervention reinforces the political resistance of powerful entities that could be targeted for blame and made to bear some burden in the solution [22]. Hence, general bias can serves as the philosophical justification for group bias. General bias is often used to protect the privilege of entities that contribute to the expansion of the obesity epidemic.

### Is general bias based on an objective judgment?

Objectivity refers to the deepest quest for discovering the true meaning of things. It also refers to the need for making true judgments and decisions which require the rejection of unjustified prejudices. Since our values are mostly inherited, objectivity is the intended cumulative product of all successful efforts to know what is true and good. Objectivity guides our mind toward what is correct and provides room for intellectual development. In turn, intellectual development offers the possibility of replacing bias with correct insights through reflection and comparison among insights [22]. The process of correction requires a renewed understanding of the fundamental causes of weight excess. Unfortunately, the obesity debate in the public sphere is dominated by preconceived views rooted in social individualism. Addressing environmental cues that foster obesity will force policymakers to address issues regarding the built environment, economic exclusion, neighborhood segregation and food availability and accessibility. The lack of objectivity in a society that stresses individual effort and self-control contradicts the findings of public health scholars. Success in confronting bias depends on society's willingness to eradicate the current epidemic of obesity.

We will begin to truly address the obesity epidemic only when a societal dialogue sustained by the political commitment to address the underlying causes of obesity is initiated. As a corrective to general and group bias, genuine dialogue calls for moral, intellectual and religious change. In moral conversion change, one commits to values beyond a mere affirmation of freedom of choice. In religious change, one relies on agapic love to prioritize values and solutions that can be used to prevent and confront obesity. In intellectual change, one relies on public health research to challenge ideologies that affect/influence citizens' perceptions and understandings of obesity. Such forms of change represent acts of transcendence through which individuals and/or groups re-examine understanding of obesity, re-think perceptions and re-structure social regard, conduct, and policies [22].

The attempt to understand obesity bias based on Lonergan's cognitive theory raises issues concerning the relevance of individualism as a framework for addressing the growing epidemic of obesity. Lonergan's approach to bias calls for a deconstruction of current perceptions of obesity. It also questions the use of individualism as a framework for assigning responsibility and conceptualizing policy.

## Ethical Assessment of Anti-Fat Bias

The major challenge that we are confronted with is to know if all judgments based on an individual ability to avert obesity are always justifiable. Since these popular judgments originate from society's dominant ideologies, how can anti-fat biases be prevented? Can we prevent it by passing anti-discrimination laws that would protect obese individuals or change public perception by reframing obesity as a public health problem?

## Anti-Fat Discrimination and Legal Protection

Discrimination against obese individuals does not necessarily promote weight loss. Instead, it may have opposite effects. Society should not tolerate any form of discriminations based on weight differential. Governments need to design anti-discriminatory laws to sanction any form of marginalization of obese people in all sectors of society. Surprisingly few legal options are available for affected individuals. Currently, there are no federal or state laws that prohibit discrimination based on weight (with the exception of the state of Michigan in the USA) [33]. There are a few local jurisdictions that address discrimination in the state of California (San Francisco and Santa Cruz) and in Washington DC [33]. Due to the lack of adequate legal dispositions, those who experience weight discrimination in the USA must seek other means to be heard and recognized. Similarly, lawyers may seek other venues to address weight-based discrimination in court. In addition to the lack of clear legal arrangements that protect obese individuals from discrimination, the food industry is still reluctant to truly change the food environment. Most food and beverages industries vocally oppose legal measures that restrict their ability to market products. The New York State Restaurant Association, for example, filed a successful legal challenge to New York City's mandate that restaurants provide calorie information on their menus [34].

The need for anti-fat discrimination legislation emerges as a requirement of justice based on the uncompromised dignity to which every human is entitled as a human being. Human dignity is not an attributed reality. Instead, it is an intrinsic reality, the kind of quality people enjoy because they are members of the human family. Human dignity is a worth that has no price, no equivalent for which the object of value can be exchanged. Consequently, whatever interferes with human worth cannot be tolerated.

A legal approach to anti-fat discrimination can indeed reduce the incidence of discrimination and protect obese individuals against the psychosocial effects of social exclusion. Although a legal approach may favor the protection of obese individuals' rights, it does not appear to be a lasting solution. Anti-discrimination laws fall into the individualistic trap by focusing solely on the protection of the rights of obese or overweight individuals. The law may prevent a member of society from ostracizing overweight individuals but will not reduce obesity risks or address structural elements that indirectly cause obesity. A purely legal solution to discrimination does not go to the core of the matter simply because it does not tackle its root causes.

Legal solutions would not really address bias which is primarily an attitude. The public may not discriminate openly against overweight or obese individuals but may develop subtle forms of discrimination as has been done to women all over the world and to black people in the USA. Laws that protect women, for example, have reduced incidence of gender-based discrimination. However, these laws have not eradicated individual or structural violence against women. Laws prohibiting discrimination may not successfully prevent anti-fat discrimination unless society reframes its understanding of obesity and addresses the deeper questions of agency, choice, responsibility, and causation.

While few reasonable people would endorse discrimination against obese individuals, banning such discrimination - for example, in hiring - is unlikely to have a serious impact on public health. Thus, theorizing obesity as a public health issue legitimates the primacy of eradication of obesity as opposed to the legal protection of obese individuals. The view that obesity is also a socio-cultural issue would reduce discrimination. This view would lead to more effective prevention policy. While reducing discrimination is a worthy sub-goal, from a public health perspective, preventing incidence of obesity and reduce its prevalence should be the ultimate goal of any comprehensive intervention.

## Reframing Risk for Obesity to Challenge General Bias

Bias restricts and distorts the decision-making processes. And when left unchecked because of the general bias against theoretical knowledge, gradual decay can incrementally drag an entire culture into lower viewpoints and restrictive horizons. The lower viewpoint is that of free choice and the restrictive horizon is that of an atomistic society. The best answer to this problem is the creation of a higher viewpoint that will address the problem at its very source. Our higher viewpoint emphasizes social interdependence, connectedness and social responsibility for health. Our higher viewpoint questions socioeconomic exclusion which results in some cases, in disproportionate distribution of obesity across population groups. This higher viewpoint provides a method for interpreting social phenomena and identifying the sources of obesity, in individuals, groups, and society as a whole. To reverse the cycle of discrimination, we suggest that obesity should be reframed within an environmental model.

Framing refers to the process of evaluating a particular public health issue for the purpose of developing policy recommendations and/or adequate solutions. Very often, in public health, the framing process uses social justice as the prism through which a health condition is understood and addressed. This process helps identify social ideologies that distort people's perception of the disease aetiology. The obesity epidemic is a social, public health, medical, public policy, and economic problem whose solution requires the involvement of all these constituencies. To bring to light all the dimensions of the obesity epidemic, a deep philosophical investigation of ideas and opinions that sustain anti-fat judgements is needed. These ideas are related to issues of causation (free choice or social production?), responsibility (social or individual?), and agency (individual or collective?). There are several different frameworks that can be employed to understand obesity: the biomedical, the individual, and the environmental or public health framework.

### Possible ways of framing

In the biomedical framework, the obese/overweight individual is perceived as addicted, with obesity being a biological problem. Obesity is understood as a polygenic disorder that can be understood, managed, and/or potentially cured using biomedical solutions [35]. Obesity is conceptualized as a result of increased calorie consumption, food choice and/or genetics. In order to design an intervention, a set of indicators that relate specifically to these determinants are measured. Solutions to obesity within this framework focus on augmenting public health education regarding the avoidance of unhealthy foods, an increase in physical activity, and the recognition of health consequences related to fat excess. While the responsibility is not placed on the individual, at the very least, his or her cooperation is required in addressing the problem of obesity. Instead, responsibility is placed on health professionals and those who are doing research on obesity. Health professionals are called upon to reinforce the willpower of individuals by providing encouragement and formal interventions to overcome addiction. Researchers are enlisted to explore the process by which addiction takes place, how overeating leads to health consequences, and which cessation programs are best for helping obese individuals to stop unhealthy eating behaviors. This frame is often supported by clinicians or pharmaceutical industries.

The perception of obesity as a medical problem overshadows its social origin. Causality is impersonal and apolitical since obesity can only be controlled through further biomedical research and not by the renewed commitment of the individual and society to act on the personal and socio-environmental determinants of this growing epidemic. The one-sidedness of this frame partially removes the burden of blame from the shoulders of the obese, but suggests a very narrow understanding of its etiology.

While an obese individual is viewed as a victim of an addiction in the previous model, the individual-behavioral framework portrays an obese person as one who has made poor choices. The biology of this model is based on the idea that excess weight is the consequence of too much food energy consumed in relation to that expended. The behavioral model does not address the genetic or socioeconomic contributors. It is up to the individual to strike the balance between food energy intake and expenditure. Such a framework places a special emphasis on the individual and his or her ability to make rational choices and have willpower to avoid becoming obese [35]. Those who make poor choices are irresponsible. Obese individuals are directly or indirectly held morally responsible for a health condition which is not always within his or her control while the behavior of food manufacturers and government regulators is largely excluded from consideration. In this frame, individualized solutions that can alter the distribution of poor choices made by individuals are emphasized. Nothing or very little is said about changes that should occur in places where obesity risks are higher. The ethics that emerges from this frame is simply one-sided. The individual-behavioral framework is not conducive to the exploration of the disproportionate exposure to and burden of harmful environmental conditions experienced by low-income and racial/ethnic minority populations in developed countries. Far from being politically neutral like the previous framework, the behavioral frame limits governmental interventions to the provision of information to individuals in order to assist them in making better lifestyle decisions. In this framework, the obese person is treated as a client, a consumer of a marketable food product, who exercises free choice and can willingly stop his or her poor eating habits if he or she wishes to do so. This framework may be endorsed by certain health professionals and lay citizens, but it is often used by market individualists and libertarian politicians to limit governmental response. A behavioral approach ascribes individual responsibility as the solution to problems that may have deeper societal roots.

Finally, the environmental or public health framework is a comprehensive framework which places lifestyle decisions and human biology in a societal context shaped by public policy choices, neighborhood segregation or deprivation, socioeconomic status (SES) differentials, unequal access to care, and market competition. Here determinants of obesity are conceptualized as economic and social relationships shaped by society's political and economic institutions. Population groups defined by these relationships are differentially helped or harmed by their position relative to each other [36]. The basic idea that sustains this framework is that relative positioning of people shapes their exposure and behaviors [36]. Individual knowledge, personal preferences, and genetic susceptibility may impact risk for obesity. However, socioeconomic and environmental factors ultimately play an important role in determining an individual's ability to prevent or manage obesity [37]. The environmental model highlights the systemic origins of obesity. It is well-established that a systemic problem cannot be solved solely by medical or individualized solutions. Obesity results essentially, though not solely, from an unhealthy food and built environment created intentionally or inadvertently by policy makers or food corporations. There is a need to shift the public discourse so prevalent in media from individual choices and responsibility to the commitment of policymakers and corporate firms to shape both the physical and market environment in such a way that they promote well-being. The shift towards a public health rather than an individual or a biomedical framework for obesity has already commenced. It can be evidenced in proposed or implemented policies to increase physical activity hours within schools, remove trans-fats from restaurant menus, enforce the display of nutritional information, increased consultation regarding appropriate urban planning, and the levying of so-called junk food taxes.

Within the public health framework, personal responsibility and the wider community responses are not contradictory. One is necessarily supported and sustained by the other. An individual's commitment to a healthy lifestyle contributes to the prevention or reduction of the burden of obesity. On the other hand, the community's commitment to create an environment that maximizes the potential for people to make healthy choices achieves the same goal. Individual choice is not only determined by the ability to exercise freedom but also by current environmental and public policy. Obesity cannot be solely addressed on the ground of autonomy, free will or personal virtue. The notion of individual choice, responsibility and autonomy is especially difficult to apply in relation to obesity. There are barriers for people wishing to achieve behavior change. People's personal behavior choices are to a substantial degree shaped by their environment, which in turn is heavily influenced by local authorities and national government, industry and others. Therefore, policies based on education, information and individual choice alone are not likely to succeed either in reducing inequalities or in reducing prevalence of obesity in the population as a whole. We do not mean to reject individual responsibility for health or undermine the efforts deployed by those who work hard to keep a healthy lifestyle. Certainly, there can be an aspect of virtue in obesity prevention. The need for controlling one's weight should be stressed as an important health promotion strategy [38]. Hence, public health strategies should aim to ensure that it is easy for people to lead a healthy life and promote health by programs to help people overcome addictions and other unhealthy behaviors. However, the call for individual responsibility cannot be strongly asserted for all persons. The call for individual behavior change should be weakened for those whose agency has been diminished by geographical, socioeconomic, or historical reasons.

Even though the public health framework highlights distal causal factors, it also includes behavioral and medical aspects of health. The inclusion of behavioral and biomedical elements that we have judged, when taken separately, to be inadequate to confront the obesity epidemic may sound contradictory. There is no contradiction as such. What we have rejected so far is the one-sided polarization on behavior or biomedical research as only solutions to the obesity epidemic. Personal responsibility and biomedical research alone do not address the distal causal factors for obesity.

### Epidemiologic theory, obesity causation and general bias

Each of these three models incorporates distinct views of etiology, prevention policy, pathology, and treatment of obesity. Each tacitly promotes different conceptions of the proper allocation of individual and social responsibility for obesity. The analysis that follows questions the use of individualism as a methodology and as an ideology. It also criticizes some basic assumptions of the biomedical model and supports the public health model as an alternative framework for analyzing obesity occurrence. Within the latter framework, our approach to epidemiological investigation takes into consideration the basic features of each of the three models: biology, lifestyle, and the environment.

The differences among our three models show that the formation of hypotheses presupposes a theoretical framework. This framework directs the interpretation of epidemiological data about obesity and largely determines what we know, how we know, what can be known and what is left aside. The biomedical model focuses essentially on biological processes while the behavioral model connects biological processes with individual behaviour or lifestyle to explain the distribution of obesity in populations. Even though the proponents of the behavioral approach acknowledge the impacts of external factors on one's risk for obesity, they emphasize the individual's ability and responsibility to master them. This view is widespread in the USA. It disregards both the dominance of powerful groups on public policy design and their influence on the process of social validation of scientific research. Considering the multiplicity of constraints on human abilities to engage in certain types of eating and activity behaviors, we need to humbly accept individuals' limitations and propose some venues for collective agency through changing eating infrastructures or the built environment. People cannot seem to effect long-term dietary changes.

A more encompassing approach, the environmental model, includes both behavioral and biological pathways that explain obesity occurrence. The environmental model presupposes the recognition of the irreducible historical situation of obese individuals both as individual persons and as social beings. Unless we clearly acknowledge the social nature of the human person, the epidemiological theory we adopt will limit the scope of our moral imagination to individualized solutions and actions. We may, then, run the risk of sounding irresponsible and socially blind because we are accountable for the knowledge we produce and its effects on the public's welfare [36]. The public health model reminds us that the work of a public health researcher has a context which is human society. It is, then, within this context shaped by social forces that distribute well-being that obesity bias should be understood and policy designed to address this growing epidemic.

## Environmental Model, Social Policy and Group Bias

Poor dietary habits and unhealthy lifestyle are not the only causes of obesity. Habits and lifestyle are often the proximal causal factors which are shaped by the interplay between many factors. These factors include social norms and values. Sectors of influence such as the food and beverage industries, media and transportation are also important distal causal factors of obesity. Lifestyle is often influenced by the social environment in which people live. These behavioral settings include home and family, school and community, and church and social networks. Individual's dietary habit may be determined by individual factors such as genetics, psychosocial, and other personal elements. In order to truly address the growing epidemic of obesity, we need to shift our focus from a clinical or psychological perspective which looks at one individual at a time to the epidemiological perspective of public health and preventive medicine. Improving public health through community design first probes how various aspects of the built environment currently contribute to obesity by affecting eating and physical activity habits and facilitating an increasingly sedentary lifestyle. Important efforts must be directed to community engagement and advocacy for policy and infrastructure change. The public health approach to obesity prevention and control is in its infancy stage. Policy initiatives and environmental changes that provide healthy choices are promising, since these same strategies have been successfully used in tobacco control [38].

The public health framework allows us to affirm that no linear progression of causes can explain the uneven distribution of obesity and of the subsequent diseases that it causes. The relationships are synergetic, and their sources remain embedded in major economic and social institutions, public policies, and infrastructural arrangements in given circumstances [39]. They are linked to historical relationships of power distribution and property ownership, systemic racism, neighbourhood segregation and poverty. Income inequality and behavior will only one-sidedly explain the clustering of obesity in a given population without investigating the links between factors such as educational opportunities, racial segregation, healthy food availability, built environment, and neighbourhood safety [40].

In order to fulfill its duty as protector of the public's health, the government can preferentially address distal causal factors of obesity without neglecting the proximal causal ones. The avenues available to play such a noble task include addressing issues related to the built environment, SES of most obese people and access to goods which are necessary to avoid obesity. Acting on distal causal factors also involves challenging the dominant market *ethos *to favor access to healthy foods and regulating the cost of food and gym facilities in order to encourage physical fitness. A prioritarian approach to reducing obesity might be considered on the basis of reducing inequalities in health. An example would be planning decisions that improve access to sports facilities or shops/markets that sell fresh fruit and vegetables, or the distribution of food vouchers to people of lower socio-economic status. However, although such targeted interventions could benefit people who might not gain from population-wide initiatives, care would be required to avoid actual or perceived stigma that may result from singling out particular social groups in this way. A prioritarian approach emphasizes social responsibility and questions socioeconomic individualism as an explanatory framework for understanding and addressing obesity.

### Beyond moral and socioeconomic individualism

Ideally, every individual should be the primary person responsible for his or her health. However, a particular manner of affirming human agency can obscure the real constraints on agency experienced by some people. Paul Farmer vehemently criticizes the tendency, prevalent in clinical practice, of blaming treatment failure and noncompliance on an individual patient while disregarding structural and operational factors that are beyond the patient's control [41]. It is precisely those persons whose agency is most constrained who are asked to improve their eating and exercising habits. Health education and individually-based solutions have been proven to be, for the most part, inefficient for the disenfranchised people living in inner cities who have no other choice but to be at risk for all sorts of diseases. Behavior is merely one factor in an environment that places people at risk. Throughout the United States, there are increased indices of economic exclusion that seem to favor epidemics in inner cities already ravaged by epidemics of AIDS, intravenous drug use, homelessness, and racism [41]. The overemphasis of agency desocializes obesity and prevents social institutions from taking responsibility for the growing burden of obesity. Moving the focus from the individual to the major players in the obesity problem would help us address the core of the matter and properly assign responsibility. Society's failure to create a better socioeconomic, food, built and political environment that may reduce incidences of obesity is clouded over.

Public health policy requires that the majority accept their fair share of the burdens of protecting a relative minority threatened with obesity. We need to ask if the prevailing model of justice legitimizes sacrifice and care for others [42]. Public health ethics is not merely an alternative to market justice. Instead, public health ethics stands as a critique of the market worldview that gives pre-eminence to moral and political individualism. Market-justice emphasizes freedom from collective obligations except those of respecting other persons' fundamental rights. On the other hand, social justice, the heart of public health ethics, requires that we work with all the social constituents to design and continually perfect our institutions as means which facilitate personal and social development [42]. The market does not always promote the goals of public health [34]. The values and beliefs of the public health community often stand in contradiction to the libertarian thinking that shapes the dominant values of the market. Advertisement to children of foods high in sugar and fat represents an example of this discord. Faced with such an assault on public health, the government needs to strengthen its regulatory role in order to protect vulnerable individuals [34].

Despite myths about individualism and self-reliance, the US government has a long tradition of regulating ostensibly private behavior [34]. This long tradition is evidenced by government exercizing its regulatory power on alcohol, illegal drugs, tobacco, and sexuality as a way to protect population health. The same regulatory power can be applied to commercialized foods and drinks. This much needed paternalistic tendency can be applied to food advertising, food pricing, and to retail competition. Very often, the justification for public health interventions is paternalistic because the goal of these interventions is to promote the well-being of those who might otherwise be inclined to certain diseases. In the USA, most people cherish their freedom to the point that some may not tolerate any form of governmental intrusion in their life. However, paternalism is often used to promote population health. This type of paternalism is not necessarily destructive of fundamental liberties. Liberty does not simply mean freedom to be left alone. Instead, liberty can be understood as a means to free society from unnecessary suffering and preventable disability and death.

Robert Goodin, a public health scholar and anti-tobacco activist, argues that the ultimate ethical justification for public health paternalism must be essentially utilitarian, turning on the way that overall social utility is maximized when the utilities of all members of the society are maximized [43]. Paternalism targets the group and its practices and not particular individuals [44]. Policies grounded in paternalism aim at institutionalizing health-promoting practices and institutions at the population level. In the other hand, paternalistic policies aim at controlling or proscribing public practices and institutions that hinder people's health and well-being. The socioeconomic and health implications of market competition bring about problems that cannot be solved by individuals. The public health consequences of food industries' practices and the burden of obesity on economy call for government intervention. To support government intervention in population health problems, we endorse Dan Beauchamp's critiques of Stuart Mill's rejection of paternalism. Beauchamp argues that "To Mill, all paternalism was wrong because the individual is best placed to know his own good" [44]. Mill is wrong because government intervention to address the underlying causes of obesity does not aim at promoting the well-being of a particular individual but of the entire community. Paternalism places (social or economic) constraints on every citizen for the good of all. The failures or limited successes of individual-based solutions tend to validate the appropriateness of structural change as the core solution to the obesity epidemic. Mill is even wrong twice because particular individuals are often very poorly placed to judge the effects that market arrangements and practices have on the population as a whole. This is the task for legislatures, for organized groups of citizens, and for other agents of the public, including the citizen as voter [44]. Paternalism operates primarily at the level of public practices and not at the level of individual behavior [44]. By intervening, the government definitely demonstrates its solidarity with low-socioeconomic and vulnerable groups [34]. Michelle Mello, a health policy scholar from Harvard University, highlights the salient place of solidarity as a means for health justice. Solidarity is the notion that individuals should not be left by their community to bear terrible burdens alone [34]. Mello's solidarity argument is philosophically defensible. The community has the moral duty to prevent suffering where its members can. Obesity is an important risk factor for chronic diseases and a shortened life span. On the solidarity ground, it can be argued that, as a representative of community, the state should intervene to prevent suffering wherever it is possible. In addition to her "solidarity argument" [34], Mello argues that "some forms of paternalism are both morally acceptable and morally laudable" because they can legitimate government intervention if a health problem affects a vulnerable population [34].

Public health emphasizes the interconnection among human beings in a democratic society as well as the importance of community mobilization to promote good health. Hence, the idea of liberty should mean, above all, the liberation of society from the injustice of preventable disability and early death. Instead, the concept of freedom has become a defence and protection of powerful vested interests, and the central issue is viewed as a choice between freedom on the one hand, and health and safety on the other [42]. Insufficient action by government in addressing socioeconomic inequalities and issues related to the built environment as a means of reducing obesity is simply unacceptable. The government's role is to build a civilization of equity [45,46]. The moral test of any society is how it treats its most vulnerable members and how those who belong to this population group are able and are encouraged to participate in decision making. Growing social inequalities and their subsequent health inequities raise questions concerning the level of social justice because the relationship that exists between poverty and obesity is not random. This relationship follows the distribution of social and public goods as well as opportunities for social mobility and health promotion across population groups. When obesity mushrooms in places where poverty is endemic and the built environment is poorly designed, the claim of justice cannot be silenced because the prevalence of obesity is rooted in structural marginalization. Lower socioeconomic groups and individuals have the most urgent moral claim on the conscience of the nation because social discrimination endangers their health and well-being. Policy interventions are needed to confront the obesity epidemic. People are called to look at public policy decisions in terms of how they affect the less fortunate members of society.

### Emphasizing social responsibility

Social justice is the core value that seeks to bring about practical and effective attention to the well-being of each member of society. It is concerned with ensuring the proper ordering of things and persons so that people experience fair treatment, a just distribution of the dimensions of well-being, a measure of control in its processes and an awareness of their worth and role in determining their own destiny. Under our approach to social justice, each person is entitled to health protection, decent housing, safe environment, access to food, basic education, participation in society's affairs and decent income because of the intrinsic dignity that every human person deserves to enjoy by the very fact of being human [42]. Individuals and local communities do not always have the power control their food and built environment. Thus, government's attempts to prevent and reduce obesity should bring about justice by providing people with a safe environment. Government's initiatives to provide communities with a safe environment not only assert the value and priority of human life over everything else but prevent vulnerable groups and individuals from acquiring this health condition.

The cost of obesity can give rise to an economic argument in favor of government intervention in the market. When individual ability to choose creates social costs borne by an entire society, government ought to intervene to stop both the human and economic costs. Michelle Mello notes that obesity generates increased health-care costs and reduced economic productivity. In terms of annual health-care costs, obesity accounts for US$75 billion, half of which is paid by the Medicare and Medicaid programs. Health-care spending on obesity accounted for more than a quarter of the rise in per-capita health-care spending from 1987 to 2011, while a 1000 employee business paid $285,000 in obesity-related health-care costs and absenteeism in 2005. These costs create a compelling case for government intervention [34].

The solution to the current epidemic of obesity requires painful costs and hard social reforms. Improving health by mitigating obesity and its related morbidity and mortality requires that the focus of society's prevention efforts be moved from the biomedical to the political and from intervention to prevention. There is a need to move from the superficial reasoning and justifications to seek an understanding of the root causes of nutritionally-related disease and death. We must deal with a host of non-biomedical factors. The limit of the biomedical approach to treatment of obesity reflects the pervasiveness of social and environmental determinants of this epidemic. To truly prevent obesity, we need to frame prevention and treatment responses in a theoretical model that integrates the principles and practices of social justice. Unless and until we deal with the distal causal factors for obesity, we will do little to prevent this epidemic. The reason we have failed is that we treated obesity primarily as a biomedical disorder, while it is rooted in socio-economic and environmental determinants. The eradication of obesity should be a societal endeavour and not simply a challenge pertaining to the individual person or the medical community.

### Addressing the social order and raising issues pertaining to socioeconomic position

The belief that to lose weight merely requires increased energy expenditure relative to energy intake is oversimplified. This belief may result in some weight loss. If the excess kilocalories leading to weight gain really is as small as some have suggested [47], then preventing and treating obesity would not be as critical a public health issue as it currently is. There is a finite limit to the amount of increased energy expenditure or decreased energy intake one can sustain. US Popular TV shows such as "Biggest Loser" demonstrate significant weight loss among obese individuals and are often used by the general public as proof that the "energy in vs. energy out" theory has validity. It follows, then, that those who do not lose weight merely lack the volition to do so. However, what is not acknowledged is that the individuals on weight loss shows such as "Biggest Loser" often have to exercise vigorously for 3 hours a day in order to achieve weight loss success [48]. This time commitment is almost impossible for the vast majority of individuals. Approximately 30 - 50% of the obesity phenotype is inherited [49]. Genes play a crucial role in body weight regulation [50]. Weight loss capability varies from one individual to another based on genes regulating food intake centrally, those affecting adipose tissue differentiation and functioning, and those impacting energy expenditure [49]. Certain medical conditions such as hypothyroidism, Prader-Willi Syndrome, and Cushing's syndrome predispose a person to significant weight gain. Interestingly, when medical reasons for weight gain are present, individuals are less likely to express anti-fat attitudes [29].

The effects of high genetic susceptibility on the risk for obesity are amplified in a high-risk environment [49]. Environmental factors such as poor dietary choice and inactivity contribute to the rising prevalence of obesity, but another important yet under-recognized environmental determinant of obesity is socio-economic status (SES) [51]. SES of a person significantly influences the purchasing power for healthy nutritious foods as well as the ability to engage in programs of physical activity. The lack of access to sufficient food for all household members at all times has been termed food insecurity. Data from the U.S. National Health and Nutrition Examination Surveys (NHANES) reveal that ~ 16% of women and ~15% of men were marginally to fully food insecure [52]. Individuals with marginal food insecurity were more likely to be obese and to gain weight compared to those from food secure households [52]. Moreover, food insecurity is associated with an increased likelihood of having diabetes [53]. Since periods of deprivation or starvation have often been followed by binge eating, [54,55] the inconsistent availability of food in food insecure households has been proposed to be one of the contributors to obesity via overconsumption of inexpensive foods with high energy density due to the person's low food purchasing power [52].

Physical activity also is most likely to be significantly impacted by SES. Individuals from low socioeconomic households may live in neighbourhoods that may not be conducive to participating in physical activity due to crime or lack of well-lit. Furthermore, those in low socioeconomic households may not be able to afford gym memberships, have access to transportation to these gyms or parks, be able to allocate time to participate in physical activity since they may be working two or more jobs, or be the head of a single-parent household. Low SES individuals may have other comorbidities such as mental health issues. Obesity under these situations may be deemed more an issue of social justice rather than the lack of virtue.

The public health framework highlights governmental and corporate responsibility as well as behavioral change in order to gain as much support as possible for necessary policy changes. The focus is not placed on food but on health. Historically, the major determinants of health are not personal choices but risks that are external to individuals. It is more likely that the risk for obesity and malnutrition will be higher in communities deprived of fresh vegetables, healthy foods and low calorie beverages than in others. Efforts to improve health status should focus on the rules, policies, and norms that define the environment. Policy is important because it is an effective means for behavior change than education alone. An effective policy change is cost effective because it can lessen or eliminate the need to continually provide remedial programs as the policy is more likely to address the fundamental causes of the problem.

### Addressing the physical and food environment: built environment and issues of access

In their population-based studies of middle-aged Finnish men, John Lynch and colleagues demonstrated a link between adult lifestyle and parental SES [56]. Those who grew up in poverty were more likely to behave poorly (smoke, eat an unhealthy diet, and not exercise) than their peers from higher income families. These results are supported by studies demonstrating obesity in adult life as linked to measures of childhood SES [57,58]. Longitudinal data reveal that those who are overweight complete fewer years of education and have lower incomes [58]. These data indicate that SES influences risks for obesity, which may in turn influence attainable SES. Obesity follows a similar social pattern as that of hunger in the 19^th ^century. Obesity is more prevalent in lower socioeconomic groups than in higher ones [59]. Only by recognizing the pervasiveness of social inequalities that fuel the socioeconomic gradient in obesity and shape both the occurrence and the distribution of obesity, we can begin to question the gravity of some assumptions that guide anti-fat discrimination. The highest rates of obesity are found among populations with the highest poverty rates and the least education. Yet, at the same time, all income and education groups are seeing steadily growing numbers of people becoming obese.

Successful control of the obesity epidemic will require changes in the environment targeting those areas where the burden of diseases caused by obesity is high [39]. Because of the cost, modifying the built environment in all risky neighborhoods may not be possible. Government should objectively define criteria that would determine which neighborhoods will benefit from such a targeted intervention and which ones would not. Provision for public goods such as a good built environment is the duty of government officials because the government has the moral obligation to protect the public's health. Similarly, provision of parks in communities and walkable neighborhoods are commonly cited as elements of the environment that promote physical activity and reduce obesity risk. Provision of these elements is beyond the reach of individuals. Instead is part of what the government should do for citizens. Justice is not served when those vulnerable to obesity-related diseases live in neighbourhoods without these amenities [60].

In the USA, for example, the built environment varies by town income. Children living in low-income towns tend to have built environments that promote energy intake and decreased opportunities for energy expenditure, thereby placing these children at increased risk for obesity [61]. Neighborhoods with low SES usually have fewer physical activity resources than medium-to high-SES neighbourhoods, leading to more inactivity of neighborhood residents. In low-SES neighborhoods, many incivilities and unsafe conditions are common, creating dangerous neighborhood environments. Residents in neighborhoods with more available physical activity resources, including sidewalks and safe streets, report higher activity levels. The proximity of these resources is important because people are more likely to use nearby resources. Making neighbourhoods more walkable might help increase physical activity [47].

Access, therefore, appears to be a relevant strategy for behavior change since it may lead to the modification of some, if not all, of the six targeted behaviors the Centers for Disease Control and Prevention point out as a way to prevent and control obesity among children [47,61]. In addition to access, overarching strategies to improve these behavioral targets include pricing, media, point-of-purchase information, and social supports and services. As a behavior change strategy, access should address both the built environment through zoning, transportation policies and the food environment. Access strategies directed at increasing physical activity and reducing sedentary lifestyles include promoting zoning and transportation policies to increase the use of public transit. Access strategies also include availability of sidewalks, parks, and mixed land use for physical activity; requiring quality daily physical activity in schools, and restricting screen time in child care settings [47]. Access strategies to improve food and its consumption include increasing the availability of supermarkets and corner stores selling healthier foods; promoting institutional procurement policies to increase healthier foods at work sites; supporting farm-to-institution programs, and limiting the availability of high-energy dense foods and sugar-sweetened beverages. Access strategies are not new. They have been implemented in Pennsylvania, New York, and Los Angeles to name but a few places. Several examples of how access strategies have been implemented exist. The Pennsylvania Fresh Food Financing Initiative funds the building of supermarkets to improve access to healthy foods in underserved areas. The Healthy Bodegas Initiative of New York City has funded local bodegas to expand the availability of healthier food choices throughout the metropolitan area. In Los Angeles, a one year moratorium on the development of new fast-food establishments within a neighbourhood of thirty-two square miles of poor and low-income residents restricts access to less healthful products by using zoning regulations [47].

### Pricing

Pricing strategies may consist of reducing or subsidizing fees at recreational facilities for physical activity. The selective pricing or even taxation of less healthy foods is another strategy that is gaining ground. Several states and local communities have started to experiment with policy initiatives that affect the built environment in an attempt to decrease the prevalence of obesity. The focus of these policy measures has generally been to eliminate geographical disparities in access to food. Recent policy proposals include the use of zoning laws to create a healthier food environment. These laws provide incentives for chain grocers to open stores in disadvantaged and underserved areas. They also provide incentives for existing food retailers to offer healthier products. The economic feasibility of implementing these types of interventions depends on the policymakers' ability to identify communities most at need [47].

### Public policy and food industry

The food industry, a major player in this epidemic, has a responsibility to confront the obesity problem. It should keep consumers nutritionally informed, create healthier options for consumers, cut portion sizes, modify marketing strategies, and develop health programs that should include making nutrition and food-related health information readily available. The food industry has already begun taking many of these actions and should continue to do so in the future. As a prime protector of the public's health, the government should continuously monitor the food industry in order to ensure that the public is truly informed about healthy nutritional choices. The government must continue to police the marketplace so as to eliminate deceptive practices. Recognizing this duty, various government agencies, such as the Food and Drug Administration (FDA) and the Internal Review Service in the USA, have already taken action. Various state legislatures, likewise, are in the process of enacting bills to stem the tide of obesity.

Segments of the food industry should take careful note of the serious lessons learned from the course of events that brought the tobacco industry to its knees. Considering obesity's current status as one of America's most serious health issues, the striking similarities to the tobacco litigation and associated public outcry related to health concerns cannot be ignored. That so many members of the food industry have voluntarily initiated changes to improve the quality of their food choices is a testament to the fact that some of the lessons hit home. These changes must continue. As medical science advances to uncover the secrets of how our bodies process food, food industries should learn from the tobacco industry's experiences and make responsible changes. The changes will, in the end, be in everyone's best interest.

## Conclusion

Far from being determined by a genuine process of knowing and judging as Lonergan instructs us through his cognitive theory, general bias, as it appears in individual and group attitudes, is more often the result of an uncritical internalization of society's stance toward overweight individuals. Not taking into account all the factors that determine obesity occurrence uncovers an insidious bias that resists insight into scientific truths. The behavioral approach is dominant in the US where the doctrines of free choice and individual autonomy are often upheld uncritically. Group bias contributes to the maintenance of the *status quo *in social order. Group bias also promotes approaches to governmental intervention in the public sphere which does not necessarily address the concern of vulnerable and most-at-risk population groups. Through the lenses of Lonergan's cognitive theory, obesity bias is both a group and general bias. Obesity biases can be overcome, not primarily by designing anti-discrimination laws to protect overweight individuals nor by launching interventions focused on changing the public's perception of weight based on psychosocial theories but by developing social policies that address the determinants of obesity. Here, we posit that the use of a reality-based epidemiologic theory can help in overcoming theoretical or general bias.

## Competing interests

The authors declare that they have no competing interests.

## Authors' contributions

JA and TJ originated the article, did the research and wrote a first rough draft of the manuscript together. TJ did the research on the epidemiological evidence of anti-fat biases and JA carried out the research on Lonergan and its use to understand anti-fat biases. JA and TJ all read the first version and contributed editorial and critical suggestions. After the first peer-review, JA made substantial revisions to the earlier draft and worked toward the final draft. They have both read and approved the final version of the manuscript.

## Authors' information

Jacquineau Azétsop obtained his PhD in social and religious ethics at Boston College, Massachusetts/USA and a Masters in Public Health (MPH) at the Bloomberg School of Public Health from Johns Hopkins University in Baltimore, USA. Currently, he is lecturer in health policy, medical deontology and bioethics at Faculté des Sciences Médicales de l'Université de N'djamena in Chad. Dr. Jacquineau has published on public health ethics and bioethics topics in Developing World Bioethics, Public Health Ethics; Philosophy, Ethics and Humanities in Medicine; and Concilium. Dr Jacquineau has also authored a book entitled: Structural violence, population health and health equity: preferential option for the poor and the bioethics of health equity.

Tisha R. Joy, MD, FRCPC

Dr. Joy is an assistant professor at the Division of Endocrinology and Metabolism, Department of Medicine of the University of Western Ontario Schulich School of Medicine & Dentistry. She has clinical interests in cholesterol disorders and type 2 diabetes and research interests in obesity, cholesterol disorders, and diabetes. She has published several articles in the fields of metabolic syndrome, body fat distribution, and cholesterol disorders. She has published several articles in these fields and authored many book chapters.
